# Global burden of antenatal depression and its association with adverse birth outcomes: an umbrella review

**DOI:** 10.1186/s12889-020-8293-9

**Published:** 2020-02-04

**Authors:** Abel Fekadu Dadi, Emma R. Miller, Telake Azale Bisetegn, Lillian Mwanri

**Affiliations:** 1 College of Medicine and Public Health, Flinders University, Health Sciences Building, Sturt Road, Bedford Park, Adelaide, SA 5001 Australia; 2 Department of Epidemiology and Biostatistics, Institute of Public Health, College of Medicine and Health Sciences, University of Gondar, Gondar, Ethiopia; 3 Department of Health promotion and Behavioral sciences, Institute of Public Health, College of Medicine and Health Sciences, University of Gondar, Gondar, Ethiopia

**Keywords:** Antenatal depression, Adverse birth outcomes, Review of reviews

## Abstract

**Background:**

Women of childbearing age are at high risk of developing depression and antenatal depression is one of the most common mood disorders. Antenatal depression is also associated with a number of poor maternal and infant outcomes, however, there remains a lack of focus on mental issues in antenatal care, particularly in lower income countries. This systematic review of reviews provides useful evidence regarding the burden of antenatal depression which may provide guidance for health policy development and planning.

**Methods:**

We searched CINAHL(EBSCO), MEDLINE (via Ovid), PsycINFO, Emcare, PubMed, Psychiatry Online, and Scopus databases for systematic reviews that based on observational studies that were published in between January 1st, 2007 and August 31st, 2018. We used the Assessment of Multiple Systematic Reviews (AMSTAR) checklist scores to assess the quality of the included reviews. We applied vote counting and narrative review to summarize the prevalence of antenatal depression and its associated factors, while statistical pooling was conducted for estimating the association of antenatal depression with low birth weight and preterm birth. This systematic review of reviews was registered on PROSPERO with protocol number CRD42018116267.

**Results:**

We have included ten reviews (306 studies with 877,246 participants) on antenatal depression prevalence and six reviews (39 studies with 75,451 participants) conducted to identify the effect of antenatal depression on preterm and low birth weight. Globally, we found that antenatal depression prevalence ranged from 15 to 65%. We identified the following prominent risk factors based on their degree of influence: Current or previous exposure to different forms of abuse and violence (six reviews and 73 studies); lack of social and/or partner support (four reviews and 47 studies); personal or family history of any common mental disorder (three reviews and 34 studies). The risk of low birth weight and preterm birth was 1.49 (95%CI: 1.32, 1.68; *I*^*2*^ = 0.0%) and 1.40 (95%CI: 1.16, 1.69; *I*^*2*^ = 35.2%) times higher among infants born from depressed mothers.

**Conclusions:**

Globally, antenatal depression prevalence was high and could be considered a common mental disorder during pregnancy. Though the association between antenatal depression and adverse birth outcomes appeared to be modest, its absolute impact would be significant in lower-income countries with a high prevalence of antenatal depression and poor access to quality mental health services.

## Background

The fifth edition of the Diagnostic and Statistical Manual of Mental Disorder (DSM-IV) defines antenatal depression as Major Depressive Episode (MDD), which mostly associated with environmental and genetic factors [1]. Childbearing age for females is the time of highest risk for developing depression and antenatal depression is one of the least investigated and under-treated disorders [2, 3]. Antenatal depression is thought to be exacerbated by a high rate of peptide and steroid hormone fluctuation occurring during pregnancy and childbearing age [4]. The prevalence of antenatal depression ranges from 7 to 20% at each trimester of pregnancy [5, 6] and longitudinal studies suggest that antenatal depression symptoms tend to persist or re-occur in subsequent pregnancies [7, 8].

Antenatal depression affects maternal quality of life and is a major cause of disease burden in both developed and developing countries; it is responsible for an estimated 6.2% of life years lived with disability [9–11]. Antenatal depression also has a high economic burden related to health service utilization estimated to reach up to 8.1 billion pounds in the United Kingdom [12] in addition to those costs associated with poorer human capital [13].

At an individual level, the risk of low birth weight, preterm birth, intrauterine growth restriction, and pregnancy complications [2, 14–20] are known to be higher in association with antenatal depression. In addition, antenatal depression has been linked to infant developmental, emotional and attachment problems, poor academic performance, malnutrition, respiratory disorders and a higher risk of the infant developing mental health disorders in later life [21–27]. Depression during pregnancy can affect maternal health seeking behavior, adherence with medical and psychological interventions and increased risk behaviors, such as that substance use and misuse [28, 29].

Reducing infant and child mortality is the primary target set for the health sector in the Sustainable Development Goals [30, 31]. The Sustainable Development Goals (SDGs) is a United Nations global initiative to end poverty, protect the planet and to ensure peace and prosperity to all global citizens by 2030 in which all the member states are pledged to achieve. Adverse birth outcomes, such as low birth weight and preterm birth, are the leading cause of infant and childhood morbidity, mortality, and neurodevelopmental impairment [18, 32, 33]. Despite the burden of preterm and low birth weight remaining high, the related risk factor of maternal mental health has not yet been a focus for prevention and control strategies set by low- and middle-income countries where 60% of births born are preterm and low weight [32–36].

The lack of policy attention on mental health problems in women of reproductive age, and the correspondingly limited number of interventions aimed at alleviating the problem in many countries might be due to a lack of comprehensive evidence. In considering published systematic reviews, decision makers could potentially be faced with a range of conclusions, and reviews that differ with respect to quality and scope. Conducting a systematic review of reviews in a logical and appropriate manner would allow for the comparison, contrasting, and production of evidence that would help policy makers and clinicians for planning appropriate and timely interventions [37] . As such, our current systematic review of reviews would have a potential usefulness for country health ministries that have suffered from inconsistent conclusions about the problem magnitude and who were unable to set intervention modalities.

## Methods

### Overview of a systematic review of reviews

A systematic review of systematic reviews, also known as an ‘umbrella review’ is a synthesis that includes only other systematic reviews, which represent the highest form of evidence. This approach aims to provide a single comprehensive source of evidence and in recent years has been increasingly used to guide policymakers and those developing intervention modalities, clinical guidelines, and in the evaluation of health care interventions [38, 39]. As with other reviews, a systematic review of reviews, follows a systematic approach in searching the literature, appraisal, quality assessment, synthesis and reporting of the compiled results [37, 40, 41].

### Search strategy and inclusion criteria for systematic reviews

We searched CINAHL(EBSCO), MEDLINE (via Ovid), PsycINFO, Emcare, PubMed, Psychiatry Online, and Scopus databases for systematic reviews based on observational studies. To include the most up to date reviews on the topic, only those published between January 1st, 2007 and August 31st, 2018 were considered. The primary outcomes of this review of reviews was the burden of antenatal depression and any associated adverse birth outcomes – specifically, low birth weight, preterm birth, and still birth.

### Outcome measures

systematic reviews that clearly measured and reported the following outcomes were included: [1] depression during pregnancy measured using a validated screening or diagnostic tool [2]; objectively measured birth weight and low birth weight was classified as a weight less than 2500 g [3]; gestation and age measured using a Last Menstrual Cycle (LMP) or supported by an ultrasound and preterm birth defined as a birth before 37 completed weeks of gestation; and [4] stillbirth defined as a fetal death after 20 completed weeks of gestation and weighing at least 500 g, intrauterine fetal death prior to the onset of labor, or intrauterine fetal death during labor and delivery.

### Example of search strategy for antenatal depression in PsycINFO via Ovid

(((antenatal depression.mp. [mp = title, abstract, heading word, table of contents, key concepts, original title, tests & measures]) OR (depression during pregnancy.mp. [mp = title, abstract, heading word, table of contents, key concepts, original title, tests & measures]))) AND (((systematic review.mp. [mp = title, abstract, heading word, table of contents, key concepts, original title, tests & measures]) OR (meta-analysis.mp. [mp = title, abstract, heading word, table of contents, key concepts, original title, tests & measures] OR (review.mp. [mp = title, abstract, heading word, table of contents, key concepts, original title, tests & measures])))

### Inclusion

reviews fulfilling the following criteria were included**:** [1] published with systematic review/meta-analysis in their title [2]; antenatal depression and its effect on birth outcomes was the primary objective [3]; systematically searched for primary studies in at least two medical literature data bases [3]; included at least one primary study that aimed to investigate antenatal depression and/or its effect on birth outcomes [5]; quality of included primary studies was assessed and considered in the analysis; and [6] if estimates in the primary reviews were meta-analyzed; the methodology, the model, publication bias, and heterogeneity issues were addressed and clearly reported.

### Exclusion

reviews were excluded if they included primary studies that screened depression in high risk populations (obese, overweight, diabetes, mothers with poor obstetric history, unintended pregnancy, primi-mothers) and reviews for which it was not possible to retrieve the full article.

### Risk of bias and data extraction

All reviews meeting the inclusion criteria were imported to an Endnote database. After duplicates were removed, titles and abstracts were assessed for eligibility prior to full text review. Reviews fulfilling the inclusion criteria through full text review were then assessed for their quality. Quality was assessed using Assessment of Multiple Systematic Reviews (AMSTAR) checklist scores. The checklist contains 11 indicators that are used to derive an overall score assessed as high quality (score > =8), medium quality (score 4–7), and low quality (score < =3). Two reviewers (AF & TA) independently assessed the quality of each review with an internal consistency of 98% and agreement was reached by discussion for the remaining 2%. The data were extracted and tabulated: author and publication year; geographic coverage of the review; data base searched; depression assessment tool used; number of primary studies included; if meta-analyses were conducted, the pooled number of participants (N); main findings; and AMSTAR score.

### Strategy for data synthesis

The data synthesis was undertaken independently for each outcome of interest. Vote counting and narrative review were used to summarize and present the main findings for antenatal depression and associated factors. Statistical pooling (meta-analysis) was conducted for quantifying the effect of antenatal depression on low birth weight and preterm birth. A funnel plot and Egger’s regression test was used to check for potential publication bias. Where minor publication bias was identified, Tweedie’s and Duval’s trim and fill analysis was used as an adjustment. Heterogeneity among the studies was tested using the Higgins method, in which *I*^*2*^ statistics were calculated and compared with the standard. The data were imported and analyzed using Stata 14 software (StataCorp. 2015. Stata Statistical Software: Release 14. College Station, TX: StataCorp LP). This systematic review of reviews was registered on PROSPERO with protocol number CRD42018116267.

## Results

### Search

We identified 230 items related to antenatal depression and 35 items related to the association between antenatal depression and birth outcomes. After duplicate removal and abstract review, 19 reviews conducted on antenatal depression prevalence and 14 reviews conducted on antenatal depression and adverse birth outcomes underwent a full text review. Seventeen reviews were excluded for the following reasons, the review/s: was a compiled report [27]; focused on perinatal depression [3, 42–44]; had non-relevant study objectives [23, 24, 45, 46]; were not systematic reviews (e.g. overviews, literature reviews or critical literature reviews) [25, 26, 47]; included primary studies conducted on high risk population [48, 49], searched only one database [50]; focused on exposures or outcomes that differed substantially from the main objectives of the current study [51] or; had no available full text [52].

**Fig. 1:**
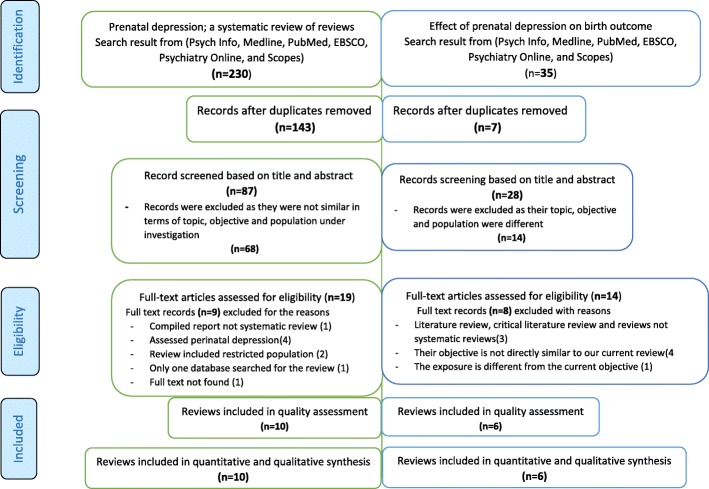
PRISMA diagram for systematic review of reviews conducted on antenatal depression and its effect on adverse birth outcomes

The remaining ten reviews (collectively consisting of 306 primary studies and 877,246 study participants) on antenatal depression prevalence [6, 46, 53–60] and six reviews (collectively consisting of 39 primary studies and 75,451 study participants) on the association between antenatal depression association and adverse birth outcomes [14–17, 61, 62] were included in the current review of reviews after assessed for quality. (Fig. 1).

### Antenatal depression prevalence and associated factors

#### Characteristics of included reviews

All included reviews were published from 2010 onwards and included only primary studies that were published from 1968 to 2017. The number of primary studies included in each of the reviews ranged from seven (with 2161 participants) to 97 (with 1,541,303 participants). Seven reviews included a majority of studies from developed countries, two reviews included only primary studies from low and middle-income countries, and one review included primary studies from only Asian countries. The predominant screening tool for measuring antenatal depression was the Edinburgh Postnatal Depression Scale (EPDS) [63], which was used by 101 primary studies across all of the reviews. PubMed/MEDLINE, Psych INFO, CINHAL and Scopus databases were the most cited data bases for searching primary studies. Four reviews reported a pooled prevalence of antenatal depression and all reviews reported risk factors associated with antenatal depression (see Table 1). Only four reviews assessed the quality of included primary studies using a standard quality assessment tool and [8] respectively, two and eight reviews fulfilled a criterion for upper and middle quality scores on AMSTAR (see Table 2).

**Table 1:** Summary of systematic reviews conducted on antenatal depression included in this systematic review of reviews (*N* = 10)

Review	Geographic coverage of the review	Prominent tools used	Data base searched	Number of primary studies	Number of participants	Quality assessment	Main findings relevant to the review		AMSTAR score
							Antenatal depression Prevalence	Risk factors	
Biaggie, 2016 From 2003 to 2015	Developed countries	Not reported	PubMed, Psych INFO, Cochrane Library	97	1,541,303	Not assessed	Not reported	- Lack of partner or social support (in 13 studies); - History of abuse or domestic violence (in 6 studies); - Personal history of mental illness (in 7 studies); - Un-planned or unwanted pregnancy (in 4 studies); - Adverse events in life and high perceived stress (in 3 studies); - Present or past pregnancy complications or loss (in 3 studies)	5
Gelaye et al., 2016 From 1998 to 2015	Low and middle-income countries	EPDS (22 studies)	PubMed, Embase, CINAHL, BIOSIS Online	51	48,904	Not assessed quality of the primary studies	25.3% (95% CI 21.4–29.6%)	- Early life abuse (child maltreatment, a severe early life stressor, includes all forms of physical, sexual and psychological maltreatment that pose harm to a child’s health, development or dignity) in 2 studies - Adult abuse (intimate partner violence (IPV), encompassing physical, psychological and sexual abuse in five studies - Maternal low educational attainment in two studies - Maternal current low economic status in three studies - Lack of social support in one study - History of mental illness in one study	7
Halim et al. 2017 From 1990 to 2017	Low and Lower middle-income countries	EPDS in ten studies	PubMed, Web of Science, Scopus, Psyc Info, Applied Social Science Index and Abstracts (ASSIA)	24	13,490	*Quality assessed but not used standard criteria*	15—65%	- Intimate partner violence during pregnancy in 24 studies	7
Mitchel et al. 2017 From 1980 to 2015	Developed countries	BDI in six studies	PubMed, MEDLINE, Embase and PsychINFO	12	4751	NOS criteria	Not reported	- Hyperemesis gravidarum in 12 studies	7
Roomruangwong C et al. 2011 From 1968 to 2010	Asian countries	BDI in six studies	MEDLINE (PubMed), PsychINFO and SCOPUS	25	9126	Not assessed the quality of primary studies	20%	- Having a history of premenstrual symptoms (in 3 studies) - Poor marital relationship (in 3 studies) - Unplanned/unwanted pregnancy especially, during premarital period (in 8 studies) - Poor obstetric history (complication before or in current pregnancy) in five studies - Financial difficulties (in 4 studies) - Lack of support from husband or relatives (in 7 studies)	4
Sparling et al. 2017 From 2008 to 2015	All studies are included from developed countries except one from India	EPDS in 21 studies	PubMed, EMBASE and CINAHL	35	88,051	Quality in Prognostic Studies tool, Cochrane Collaboration tool	Not reported	- 173 studies, including three polyunsaturated fatty acids (PUFA) supplementation trials, found no evidence of an association between polyunsaturated fatty acid and depression) - 22 studies showed protective effects of healthy dietary patterns, multivitamin supplementation, fish and PUFA intake, calcium, vitamin D, zinc and possibly selenium from depression. - Given the methodological limitations of existing studies and inconsistencies in findings across studies, the evidence on whether nutritional factors influence the risk of perinatal depression is still inconclusive.	7
Underwood et al. 2016 From 2000 to 2015	Developed countries	EPDS in 13 studies	Embase, PsychINFO, MEDLINE and Cochrane Reviews	16	35,419	List of criteria’s that could resemble the standard quality assessment tool	17%	- Previous depression history was found as a predictor of a current depression during pregnancy in five studies	7
Wosu Ac et al. 2015 From 1999 to 2014	Majority were from USA but the rest were from other developed countries	CES-D/ in three studies	PubMed, EMBASE, PyscINFO, CINAHL, Web of Science, BIOSIS, and Science Direct	7	2161	Newcastle-Ottawa Scale (NOS)	Not reported	- Childhood sexual abuse is strongly associated with prenatal depression (six studies)	8
Lancaster CA et al. 2010 From 1980 to 2008	Developed countries	CES- D in 49 studies	PubMed, CINAHL, SCOPUS, PsycINFO, Sociological Abstracts, ISI Proceedings, ProQuest	57	36,257	Quality assessment tool adapted from methods of the US Preventive Services Task Force	Not reported	- Life stress (in 18 studies), - lack of social support (in 24 studies), - Domestic violence (in seven studies) - Unwanted pregnancy (in six studies) - Lowe income (in 11 studies) - Unemployment (in 14 studies) - Lower education (in 20 studies) - Smoking (in 11 studies) - Alcohol use (in 10 studies) - Illicit drug use (in 8 studies) - Nulliparity (in 18 studies) - Poor obstetric history (in 10 studies)	7
Howard LM et al. 2013 From 2000 to 2012	From all continent except Africa	EPDS in 35 studies	Medline, Embase, and PsycINFO, and hand searches of Trauma Violence and Abuse, Journal of Traumatic Stress, and Violence Against Women	67	171,465	Yes, quality appraisal checklist	Not reported	- Life time domestic violence (in 11 studies) with a pooled odds ratio and 95%CI [3.04: 2.31,4.01, I^2^ = 51.1%] - Any past year partner violence in five studies, Pooled odds ratio with 95%CI [2.82: 1.52, 5.28, I^2^ = 75.3%] - Partner violence during pregnancy in seven studies, Pooled odds ratio with 95%CI [5.00: 4.94, 6.17, I^2^ = 23.7%]	8

**Table 2:** Summary of risk factors associated with antenatal depression (*N* = 10)

Risk factors	Number of reviews in which the risk factor was reported	Number of primary studies in which the factor was reported	Total participants
History of abuse (childhood or current sexual, physical or psychological) or domestic violence or intimate partner violence	6	73	293,621
Lack of partner or of social support and poor marital relationship	4	47	226,078
Personal or family history of any mental disorder or stress	3	34	177,014
Un-planned or unwanted pregnancy specially during premarital condition or nullparity	3	36	70,296
History of poor obstetric condition like current or past pregnancy complications such as hyperemesis gravidurum, adverse birth outcomes (low birth weight, preterm, still birth or infant lose after delivery), had cesarean section delivery	4	33	56, 916
Maternal low economic status or unemployment condition or financial difficulties	3	32	20,239
Maternal poor behavioral condition or practices like smoking, alcohol use, illicit drug use)	1	29	18,444
Maternal low educational status	2	22	14,638
	10 reviews	306 primary studies	877,246 participants

### Findings

As is presented in Table 1, antenatal depression prevalence ranged from 15 to 65% [54] and, among reviews reporting a pooled prevalence, antenatal depression prevalence in low and middle-income countries was higher than in high-income countries.

Psychosocial factors were the most common risk factors for antenatal depression identified across all reviews. Current or previous exposure to different forms of abuse and violence was associated with antenatal depression in six reviews of a total of 73 primary studies (collectively including over 290,000 pregnant mothers). Lack of social and partner support was the next most commonly associated risk factor for antenatal depression as reported in four systematic reviews, encompassing 47 primary studies (around 226,000 study participants). Personal or family history of any common mental disorder was the third most reported risk factor, reported in three reviews and 34 primary studies (involving around 177,000 study participants in total).

Other variables commonly associated with antenatal depression were related to maternal obstetric and economic factors. Unplanned or unwanted pregnancy significantly increased the risk of antenatal depression and the risk was much higher in premature or nulliparous mothers in three reviews involving 36 primary studies (more than 70,296 participants). Lower economic status or financial difficulty also increased the risk of antenatal depression in three reviews of 32 primary studies (more than 20,000 pregnant mothers). Having a history of poor obstetric outcomes, such as past pregnancy complications (hyperemesis gravidarum, cesarean section, hypertension, diabetes mellitus), adverse birth outcomes (low birth weight, preterm birth, stillbirth, abortion), and infant loss after birth was also associated with increased risk of antenatal depression in four reviews of 33 primary studies (around 57,000 pregnant mothers).

Education level and lifestyle factors were also associated with an increased risk of antenatal depression (see Table 2). Pregnant mothers with a history of smoking, alcohol and illicit drug use were significantly associated with depression in one review of 29 primary studies (approximately 18,000 participants). In two reviews (of 22 primary studies and more than 14,000 mothers,) low educational status was associated with increased risk the development of antenatal depression. One review conducted to test the role of diet and nutritional supplementation on antenatal depression reported inconclusive findings [59]. (Table 3).

**Table 3:** AMSTAR score of included reviews on antenatal depression and associated factors (N = 10)

AMSTAR criteria	Name of the reviews									
	Biaggie, 2016	Gelaye, 2016	Halim, 2017	Mitchel,2017	Roomruangwong, 2011	Sparling, 2017	Underwood, 2016	Wosu, 2015	Lancaster,2010	Howard, 2013
1. Was a-priori design provided?	Yes	Yes	Yes	Yes	Yes	Yes	Yes	Yes	Yes	Yes
2. Was there duplicate study selection and data extraction?	No	No	Yes	Yes	No	Yes	Yes	No	Yes	Yes
3. Was a comprehensive literature search performed?	Yes	Yes	Yes	Yes	Yes	Yes	Yes	Yes	Yes	Yes
4. Was status of publication (e.g. grey literature) used as an inclusion criterion?	Yes	Yes	Yes	No	No	No	No	Yes	No	No
5. Was a list of studies (included and excluded) provided?	Can’t answer	Can’t answer	Can’t answer	Can’t answer	Can’t answer	Can’t answer	Can’t answer	Can’t answer	Can’t answer	Can’t answer
6. Were the characteristics of included studies provided?	Yes	Yes	Yes	Yes	Yes	Yes	Yes	Yes	Yes	Yes
7. Was the scientific quality of the included studies assessed and reported?	No	No	Can’t answer	Yes	No	Yes	Yes	Yes	Yes	Yes
8. Was the scientific quality of the included studies used appropriately in formulating conclusions?	No	No	Can’t answer	No	No	No	No	No	Yes	No
9. Were the methods used to combine the findings of studies appropriate?	Yes	Yes	Yes	Yes	Yes	Yes	Yes	Yes	Yes	Yes
10. Was the likelihood of publication bias assessed?	Not applicable	Yes	Not applicable	No	Not applicable	Not applicable	Not applicable	Yes	Not applicable	Yes
11. Was the conflict of interest stated?	No	Yes	Yes	Yes	No	Yes	Yes	Yes	No	Yes
Total AMSTAR score	5 (middle)	7(middle)	7(middle)	7(middle)	4(middle)	7(middle)	7(middle)	8(upper)	7(middle)	8 (upper)

### Association of antenatal depression with adverse birth outcomes

#### Characteristics of included reviews

We identified six reviews that investigated the effect of antenatal depression on birth outcomes, with preterm birth and low birth weight as the main adverse outcomes reported. The systematic reviews were published from 2010 onwards and included primary studies that were published from 1977 to 2015 that were conducted in developed countries. The Center for Epidemiological Depression Scale (CED-S) [64] was the most commonly used screening tool in the reviewed primary studies (used by 52 studies), among which 25 primary studies investigated the association between antenatal depression and low birth weight and 39 investigated the association between antenatal depression and preterm birth. Three of the six reviews included a meta-analysis (see Table 4). Four reviews fulfilled the higher quality criteria of the AMSTAR assessment and the remainder were scored in the mid-range (see Table 5).

**Table 4:** Characteristics of studies included in a systematic review of reviews for assessing the effect of prenatal depression on birth outcomes (N = 6)

Author, year	Geographic coverage of the review	Prominent tools used	Database searched	Number of studies and number of participants	Quality assessment tool used	Main findings relevant to our review		AMSTAR score
						PTB	LBW	
Ascort et al. 2014 Included studies conducted in the year from 1977 to 2013	USA, Europe, Asia	CES-D (19 studies)	PubMed and PsycINFO	- PTB = 50 studies with Sample size of 286,043 - LBW = 33 studies with sample size of 43,534	NOS	- Taking into account the methodological quality of studies, research on depression and PTB/GA is inconclusive at best - Is a systematic review as pooling was not conducted	- It was found that about more than half (53%) of published LBW findings reported statistically significant associations with prenatal depression and low birth weight - Was a systematic review as pooling was not conducted	8
Araujo et al. 2010 Included studies conducted in the year from 1996 to 2007	United States, Norway, Canada, Denmark, India, England, United Kingdom	Not found	PubMed, SciELO, and ISIWEB u	10 studies with sample size of 231,201	Downs & Black quality assessment check list		Depression during pregnancy was associated with low birth weight in seven studies - Was a systematic review as pooling was not conducted	5
Grigoriadis S et al. 2013 Included studies conducted in the year from 1992 to 2010	most of the studies were in USA while few share Australia, China, Europe	CES-D at different cut of value in 11 studies	MEDLINE, EMBASE, CINAHL, Scopes and PsycINFO	- PTB = 15 studies with sample size of 23,754 - LBW = 6 studies with sample size of 14,090	Systematic Assessment of Quality in Observational Research (SAQOR) and the Newcastle-Ottawa Scale	- Preterm birth (PAOR = 1.37; 95% CI, 1.04 to 1.81) - I^2^ = 61% - No evidence of publication bias	- Low - birth weight was not significantly associated with prenatal depression (POR = 1.21; 95% CI, 0.91 to 1.60) - I^2^ = 0.0% - No evidence of publication bias	10
Grote K et al. 2010 Included studies conducted in the year from 1980 - 2009	most of the studies were from USA while the rest are from Europe, Asia, Brazil	CES-D in 10 studies	MEDLINE, PsycINFO, CINAHL, social work abstracts, social services abstracts, and dissertation Abstracts international databases	- PTB = 20 studies with sample of 29,295 - LBW = 11 studies with sample size of 13,544	Developed by modifying the instrument by Downs and Black	- Preterm birth is associated with depression during pregnancy PRR = 1.39 [1.19–1.61] - The association between antenatal depression and risk of PB was found to be higher in among women of lower socio-economic status in the United States. - I^2^ = 61% - Publication bias checked and corrected	- Low Birth Weight was associated with depression during pregnancy PRR = 1.49 [1.25–1.77] - The association between antenatal depression and risk of LBW was found to be higher in developing countries as compared to USA (RR = 2.05; 95% CI, 1.43–2.93) - I^2^ = 70% - Publication bias checked and corrected	7
Jarde A et al. 2016 Included studies conducted in the year from 1992 to 2015	most of the studies were from USA and other developed countries	DSM-IV (nine studies and CES-D (six studies)	MEDLINE, EMBASE, PsycINFO, cumulative index to nursing and allied health, Cochrane Central Register of Controlled Trials, and Web of Science.	- PTB = 14 studies - LBW = 8 studies with sample size of 25,663	Newcastle-Ottawa Scale	Antenatal depression was associated with an increased risks of preterm birth (odds ratio [OR], 1.56; 95% CI, 1.25–1.94) - I^2^ = 39%	Antenatal depression was associated with an increased risk of low birth weight (OR, 1.96; 95% CI, 1.24–3.10) - I^2^ = 48%	10
Staneva A et al. 2015 Included studies conducted in the year from 1992 to 2015	The majority of studies (27) were from the USA, the remaining are from Europe, Brazil (2), Canada (1), UK (1), Norway (1), and China (1)	CES-D in six studies	MEDLINE, CINHAL, PsycInfo, and Cochrane databases and manual searches were performed through reference list of included studies	- 14 studies investigated the association between antenatal depression and PTB - Sample size not clear	checklist developed by a knowledge synthesis group for the specific purpose of review of the evidence relating to determinants of preterm birth and low birth weight	- Preterm birth was independently and significantly predicted by antenatal depression - Was a systematic review as pooling was not conducted		7

Note: *PTB* Preterm birth *LBW* Low birth weight *PRR* Pooled relative risk *POR* Pooled odds ratio *RR* Relative risk *OR* Odds ratio

**Table 5:** Summary for reviews included in effect of antenatal depression on birth outcomes, a systematic review of reviews (*N* = 6)

Type of outcome	Number of primary studies	Sample size include	Estimates from review	Pooled estimates, I^2^
Low birth weight	6	14,090	PAOR, (1.21; 95%CI: 0.91, 1.60)	PAOR = 1.49 (95%CI: 1.32, 1.68) I^2^ = 0.0%(*P* = 0.213) - No evidence of publication bias - No influential study found
	11	13,544	PRR = 1.49(95%CI: 1.25, 1.77)	
	8	25,663	PAOR = 1.96(95%CI: 1.24, 3.10)	
Total	25	53,297		
Preterm birth	15	23,754	PAOR, (1.37; 95%CI: 1.04, 1.81)	PAOR = 1.40 (95%CI: 1.16, 1.69) I^2^ = 35.2%(*P* = 0.771) - No evidence of publication bias - No influential study found
	20	29,295	PRR = 1.39(95%CI: 1.19, 1.61)	
	14		PAOR = 1.56(95%CI: 1.25, 1.94)	
Total	39	75,451		

Note: *PAOR* Pooled adjusted odds ratio *PRR* Pooled relative risk

### Findings

Four of the five reviews investigating low birth weight reported an increased risk [14, 15, 17, 61] among mothers with antenatal depression and one review reported no association [16]. In regard to preterm birth, four of the five reviews focusing on this outcome reported that antenatal depression increased the risk [15, 16, 61, 62] and one reported non-conclusive findings [17]. (Table 6).

**Table 6:** AMSTAR score of included reviews for effect of antenatal depression on adverse birth outcomes (N = 6)

AMSTAR criteria	Name of the reviews					
	Accort, 2015	Araujo, 2010	Grigoriadis, 2013	Grote, 2010	Jarde, 2016	Staneva, 2015
1. Was a-priori design provided?	Yes	Yes	Yes	Yes	Yes	Yes
2. Was there duplicate study selection and data extraction?	Yes	No	Yes	Yes	Yes	Yes
3. Was a comprehensive literature search performed?	Yes	Yes	Yes	Yes	Yes	Yes
4. Was status of publication (e.g. grey literature) used as an inclusion criterion?	No	No	Yes	No	Yes	No
5. Was a list of studies (included and excluded) provided?	Can’t answer	Can’t answer	Can’t answer	Can’t answer	Can’t answer	Can’t answer
6. Were the characteristics of included studies provided?	Yes	Yes	Yes	Yes	Yes	Yes
7. Was the scientific quality of the included studies assessed and reported?	Yes	Yes	Yes	Yes	Yes	Yes
8. Was the scientific quality of the included studies used appropriately in formulating conclusions?	Yes	No	Yes	No	Yes	No
9. Were the methods used to combine the findings of studies appropriate?	Yes	Yes	Yes	Yes	Yes	Yes
10. Was the likelihood of publication bias assessed?	Not applicable	Not applicable	Yes	Yes	Yes	Not applicable
11. Was the conflict of interest stated?	Yes	No	Yes	No	Yes	Yes
Total AMSTAR score	8 (upper)	5(middle)	10 (upper)	7(middle)	10(upper)	7(upper)

**Fig. 2:**
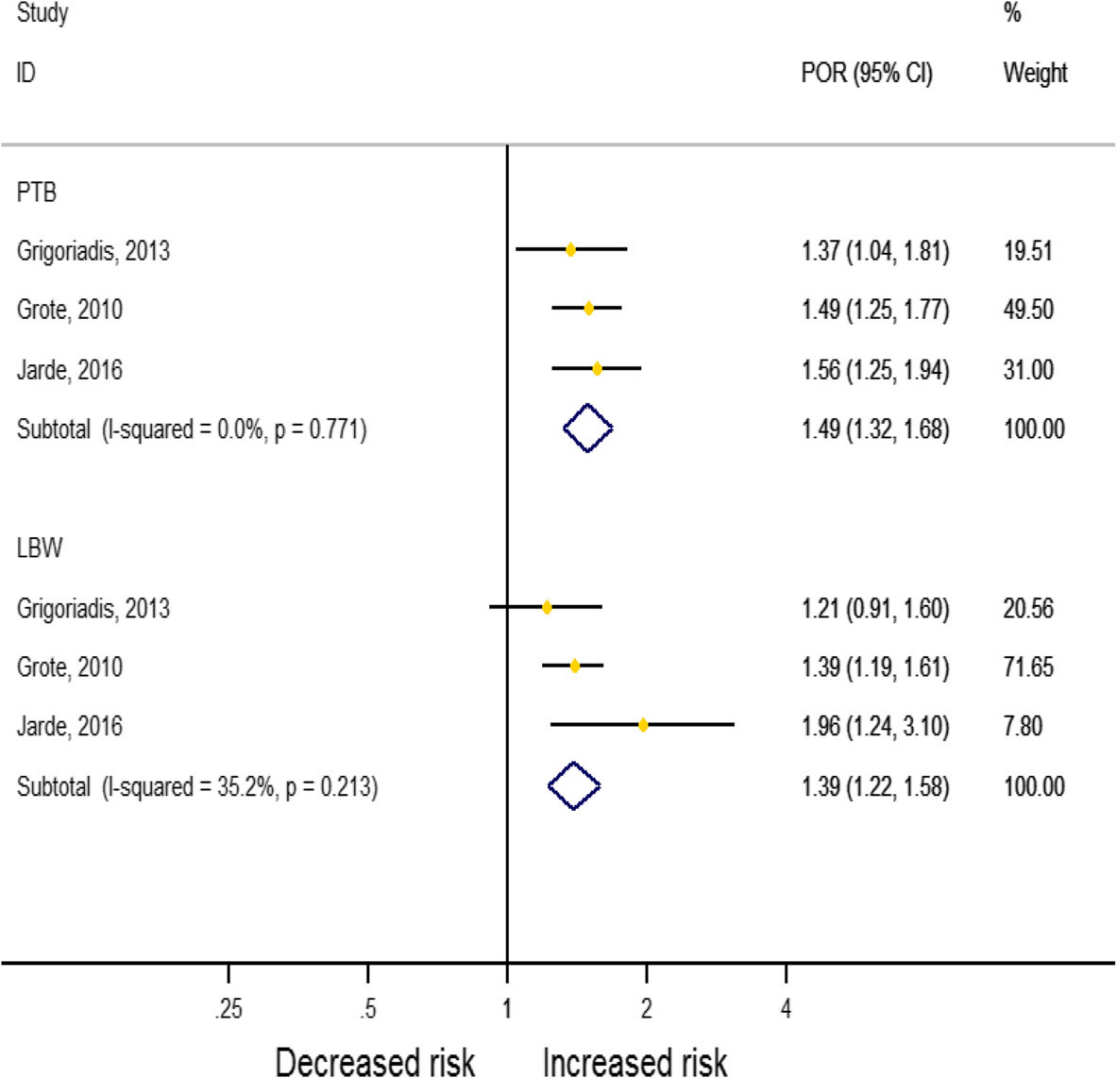
Association between antenatal depression, low-birth weight and preterm birth in systematic reviews

By pooling the estimates of three reviews (see Table 3), we estimated that the risk of preterm birth and low birth weight was 1.49 (95%CI: 1.32, 1.68; *I*^*2*^ = 0.0%) and 1.39 (95%CI: 1.22, 1.58; *I*^*2*^ = 35.2%) times higher among pregnant mothers with antenatal depression, respectively. Our test of publication bias confirmed no evidence of missing studies, and the results of our sensitivity analysis indicated that no study unduly influenced the pooled estimate (see Table 6 and Fig. 2).

## Discussion

Historically, there has been little preventive effort to address antenatal depression in many countries, despite the associated risks for adverse pregnancy and birth outcomes [7, 65]. It is possible that this may be due to limited conclusive information available about the disorder. We conducted this systematic review of reviews to comprehensively summarize the global burden of antenatal depression and its consequences on birth outcomes. We found antenatal depression prevalence ranged from 15 to 65% based on ten identified systematic reviews, themselves based on 306 primary studies. Antenatal depression was identified as a risk factor for low birth weight and preterm birth when estimates from six systematic reviews (based on 64 primary studies) were summarized.

Four systematic reviews reported a pooled prevalence of antenatal depression [6, 46, 54, 58], from which two were included studies from low and middle income countries [46, 54] while only one review included studies from high income countries [6]. A pooled antenatal depression prevalence of 17% was found in a review conducted in developed countries while a prevalence range of 15–65% was reported in a review conducted by including studies from low-and middle-income countries. All systematic reviews conducted by including primary studies from developed countries and low- and middle-income countries revealed a significantly high burden of the disorder, implying that it should be considered a major public health problem during pregnancy. Our finding supported that depression disorder was a significant cause of disease burden globally as also clearly indicated in the Global Burden of Disease studies [11, 66].

Depression is relatively common across the population but is known to be more prevalent in females relative to males [18, 67–70]. Risk for depression in females doubles during pregnancy [71], which is thought to be due to a mixture of hormonal changes and a range of psychosocial factors [72–75] that may continue to impact on mental health throughout the lifespan [74].

Given the primary caregiving role that women often have, antenatal depression may ultimately have significant implications for child development [76]. It has been proposed that maternal depression could have an intergenerational effect as female born to depressed mothers were two times at risk of having perinatal depression relative to those born to non-depressed mothers [77, 78]. In females whose mothers experienced depression, signs of depression have been noted at age 20 (38%) and 35 years (65%) [79], which might be explained by ‘fetal programming’ [80]. The negative impacts of maternal depression on child development have been identified as physical, behavioral, social, emotional and cognitive [81, 82]. Maternal depression may also result in poor intrauterine growth, miscarriage, and other adverse maternal and birth outcomes that could lead to increased infant morbidity and mortality [23]. Despite these impacts, to date, no routine maternal depression interventions have been implemented globally [67, 83, 84].

Although six systematic reviews reported history of abuse or violence as a risk factor of antenatal depression [56, 58], from considering the information across a number of systematic reviews, our study is the first to identify a history of abuse or violence as the principal risk for antenatal depression. A number of experimental studies explain the biological mechanisms underlying the association between life time abuse and violence and later depression. The alteration of brain morphology and function [85, 86], hormonal fluctuations and high concentration of corticotrophin releasing hormone, and cortisol production from hypothalamic adrenal pituitary were associated with exposure to early life adversity [87, 88]. Previous studies have observed inflammatory and epigenetic pathways in people with depression symptoms [89, 90] and past memories of being abused and violated may increase risk of later depression [91, 92].

We found that reduced or absent social or partner support was the second most replicable risk factor for antenatal depression. Social and partner support during pregnancy is highly important as it can play a buffering role and enhance coping ability and emotional stability [93, 94]. In contrast, lack of social support has been noted to enhance feelings of worthlessness and hopelessness [95].

Unplanned pregnancy and history of obstetric complication were also identified as important predictors of antenatal depression. Perceptions of increased economic burden and reduced ability to cope potential societal stigma may increase the risk of developing stress or depression [58, 96]. Adverse pregnancy and birth outcomes are often traumatic events, and a history of such complications may increase levels of stress during later pregnancy [19, 48, 53].

Socio-economic and behavioral determinants such as financial difficulties (reported in three reviews of 32 primary studies), lower educational status (reported in two reviews of 22 primary studies) and health compromising behaviors (reported in one review of 29 primary studies) were also found to increase the odds of depression during pregnancy.

We confirmed that antenatal depression increased the risk of preterm birth and low birth weight. The causal mechanism between antenatal depression and adverse birth outcomes has been well established and could be explained both genetically and socio-environmentally. Hormonal dysregulation, antenatal depression and/or chronic stress lead to changes in hypothalamic pituitary adrenal axis (HPA) function. This results in stimulation of high cortisol production and release that can restrict flow of nutrients and oxygen to the fetus [97–100]. Antenatal depression may also affect maternal immune system function via glucocorticoid hormone imbalance that may increase susceptibility to various microbial infections [101–104] and poor fetal growth. In relation to socio-environmental factors, antenatal depression may reduce capacity to access maternal health services, while potentially increasing reliance on risk behaviors such as poor nutrition (under [105–108] or over nutrition) [109–111].

To our knowledge, this is the first systematic review of reviews examining antenatal depression and adverse birth outcomes published to date. Using a systematic approach to systematically review 16 high quality reviews, which collectively reviewed over 300 primary studies, has now provided a comprehensive compilation of relevant evidence on which to base effective health policy.

It is important, however, to consider some of the limitations of our study that may have affected our results. Although the use of validated screening tools among the primary sources reviewed by the studies included in our review form part of our quality assessment, the use of different validated depression screening tools with different cutoff values may have introduced some heterogeneity, as would the use of different study design among primary studies. Although we closely scrutinized the primary studies for duplication, there remains the potential for versions reporting the same primary study to have been included in multiple reviews, which is a known limitation of the systematic review of reviews method. Given the majority of included reviews were conducted in higher income countries, reviews from low income countries were therefore underrepresented.

## Conclusions

Our systematic review of reviews confirmed that there is a high prevalence of antenatal depression in the world and a particularly high prevalence in low income countries relative to high income countries. Whilst the association between antenatal depression and adverse birth outcomes appeared modest, its absolute impact would be significant in lower-income countries with a high prevalence of antenatal depression and poor access to quality mental health services. Antenatal mental health screening has been established in higher income countries but low- and middle-income countries, who shoulder a higher burden of antenatal depression, lags far behind in implementing any intervention and management measures. Based on our findings, it is important to increase the focus on antenatal depression screening in order to address the largely avoidable adverse impacts on maternal and infant outcomes.

## Data Availability

All data generated or analyzed during this review are included in this manuscript.

## Abbreviations

AMSTAR: Assessment of Multiple Systematic Reviews
AND: Antenatal Depression
CED-S: Center for Epidemiological Depression Scale
DSM-IV: Diagnostic and Statistical Manual of Mental Disorders
EPDS: Edinburgh Postnatal Depression Scale
LMP: Last Menstrual Cycle
MDD: Major Depressive Episode
WHO: World Health Organization
